# Thyroid storm complicated by metabolic encephalopathy in a young patient: A case report and literature review

**DOI:** 10.1097/MD.0000000000046364

**Published:** 2025-12-12

**Authors:** Bixiu Ban, Yuping Liu, Zuojie Luo

**Affiliations:** a Department of Clinical Nutrition, The First Affiliated Hospital of Guangxi Medical University, Nanning, Guangxi, People’s Republic of China; b Department of Endocrinology, The First Affiliated Hospital of Guangxi Medical University, Nanning, Guangxi, People’s Republic of China.

**Keywords:** hyperthyroidism, metabolic encephalopathy, rare disease, thyroid storm, Wernicke encephalopathy

## Abstract

**Rationale:**

Metabolic encephalopathy is a rare but potentially devastating complication of thyroid storm. Its etiology is complex, clinical manifestations are diverse, there is no clear diagnostic criteria, so it is easy to lead to missed diagnosis, misdiagnosis, and mistreatment. We report a case of thyroid storm complicated by metabolic encephalopathy.

**Patient concerns:**

A 20-year-old male patient with a history of hyperthyroidism was admitted to the hospital because of limb twitching, foaming at the mouth and coma. The laboratory test results confirmed the diagnosis of thyroid storm. After treatment, the patient’s clinical symptoms gradually improved.

**Diagnosis:**

The diagnosis was thyroid crisis complicated with metabolic encephalopathy, sepsis, pneumonia, acute kidney damage, liver function impairment, and rhabdomyolysis.

**Interventions:**

The patient was given 100 mg hydrocortisone intravenously per 8 hours, propylthiouracil tablets of 250 mg q4h by nasal feeding, propranolol of 50 mg q4h by nasal feeding, piperacillin 5 g q8h intravenously, tigecycline 50 mg q12h (the first drug is 100 mg) intravenously, voriconazole 0.2 g q12h intravenously, and continuous kidney replacement therapy.

**Outcomes:**

The patient’s vital signs stabilized, his consciousness level improved, and after being taken off the ventilator, he was transferred to the Department of Traditional Chinese Medicine, where he received Chinese medicine and rehabilitation treatment, and was discharged from the hospital after his condition improved.

**Lessons:**

Thyroid storm complicated by metabolic encephalopathy requires early diagnosis and treatment, and the neurological function damage caused by it is usually reversible.

## 1. Introduction

Thyroid storm is a life-threatening emergency caused by the sudden increase of free thyroid hormone in the body or the overreaction of the body to hormones. The disease progresses rapidly, and serious complications such as shock, heart failure, and even decompensation of multiple organs may occur. The case fatality rate can reach 8% to 25%.^[1–3]^ Thyroid storm can be triggered by many other conditions, including sudden disuse of anti-hyperthyroidism drugs, trauma, surgery, childbirth, severe infections, and intense emotional distress. Common signs and symptoms of thyroid storm include a sudden surge in body temperature, heavy sweating, rapid heart rate and even atrial fibrillation, hypertension, vomiting, diarrhea, dehydration, and changes in mental status. To our knowledge, there have been few reports of metabolic encephalopathy associated with thyroid storm.^[4–7]^

As we all know, there are many forms of thyroid encephalopathy, such as Hashimoto encephalopathy, acute thyrotoxic tyopathy and metabolic encephalopathy.^[8–10]^ Among them, metabolic encephalopathy is a change in the environment of the brain tissue caused by impaired biochemical metabolism in the body, which leads to changes in consciousness caused by diffuse or whole brain dysfunction, but there is no primary structural dysfunction.^[11,12]^ Metabolic encephalopathy is a rare but potentially devastating complication of thyroid storm. Its etiology is complex, clinical manifestations are diverse, and there is no clear diagnostic criteria, so there is not enough understanding of this disease in practical work, and it is easy to miss diagnosis and misdiagnosis, which affects the prognosis and is not conducive to the secondary prevention of patients. Early diagnosis and treatment are very important for the prognosis of thyroid storm complicated by metabolic encephalopathy.

Here we report the case of a patient with thyroid storm complicated by metabolic encephalopathy and review the literature on the clinical, radiological, and prognostic features of thyroid storm complicated by metabolic encephalopathy.

## 2. Case presentation

A 20-year-old man was brought to the emergency room of our hospital with convulsions for >9 hours. The patient’s parents complained that the patient’s limbs twitched, foaming at the mouth, eyes turned up, and was unconscious before admission, which lasted for about 10 minutes. On admission, he had a body temperature of 39.3 °C, pulse of 168 bpm, blood pressure of 94/38 mm Hg, pulse oxygen of 94%, and a respiratory rate of 35 bpm with shallow breathing. Upon admission, he remained irritable, perspired profusely, vomited a large amount of stomach contents once, approximately 600 mL in volume, and the limbs involuntarily contorted. Temporarily give symptomatic treatment such as sedation, anti-epilepsy, heart rate control, fluid replacement, and oxygen inhalation. On detailed history taking, we were informed that the patient was found to have a deficiency in glucose-6-phosphate dehydrogenase at birth. When he was 1 year old, he developed convulsions in the limbs, accompanied by foaming at the mouth and fever. The duration was unknown, and no specific treatment was administered at that time. When he was 18 years old, he was diagnosed with hyperthyroidism, and his parents complained that he had been taking 2 tablets of propylthiouracil 3 times a day for anti-hyperthyroidism and metoprolol for controlling the ventricular rate, but he has stopped taking the medication on his own for 2 months. The patient had no family history of thyroid, heart, or neurological diseases. The patient had no history of smoking or drinking. This study was approved by the hospital’s ethics committee, and written informed consent was obtained from the patient for all diagnostic and treatment procedures.

Physical examination revealed that the patient was comatose with a score of 6 in the Glasgow Coma Scale (Eye = 1, Verbal = 2, Motor = 3), a score of 20 in the Acute Physiology and Chronic Health Evaluation II and a score of 7 in the Sequential Organ Failure Assessment. He was sweating profusely. His palpebral fissures were equal in size, without ptosis, and his pupils were equal in size and round, about 1.5 mm in diameter. His direct and indirect light reflexes were dull, and there was no nystagmus. The goiter was II° enlarged and soft, with obvious vascular murmurs. The breathing sound of both lungs is coarse, and a small amount of wet rales can be heard in both lungs, while dry and wet rales are not heard. The patient had tachycardia, rhythmic consistency, S1 hyperactivity, and no murmurs were heard in the auscultation area of each valve. His limbs were cold, and the femoral artery shot could be heard. Muscle tone in the extremities is normal. Deep and shallow reflexes are normal. Pathological reflex and meningeal irritation were negative.

**Figure 1.**
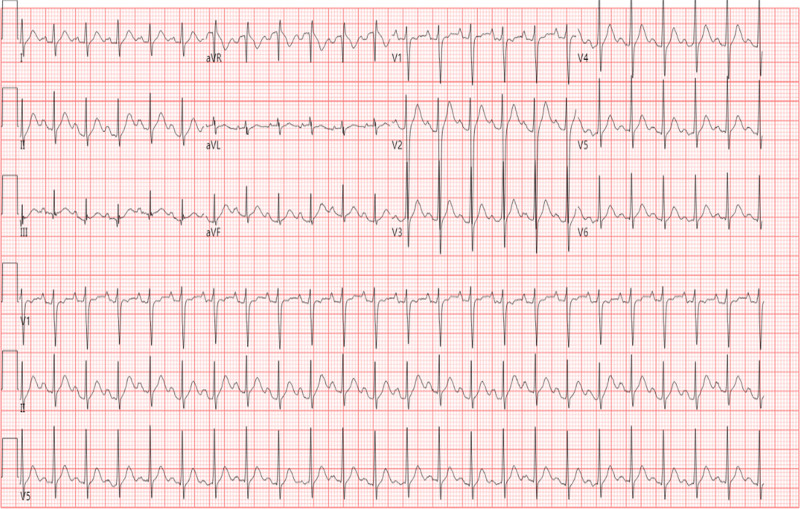
Electrocardiogram (EKG) on admission showed sinus tachycardia (heart rate: 139 bpm).

**Figure 2.**
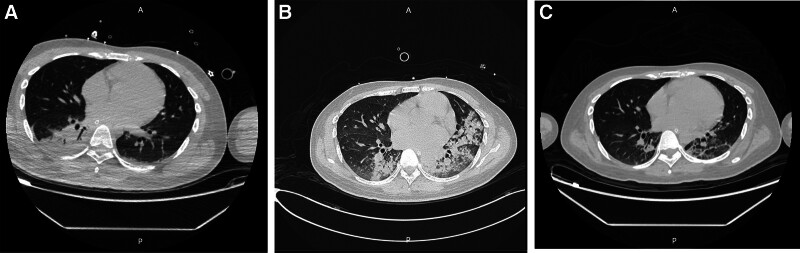
(A) Computed tomography (CT) of the chest on admission revealed bilateral pneumonia, incomplete expansion of the right lower lobe, and a small amount of pleural effusion on both sides. (B) Chest CT on the second week of admission showed that bilateral pneumonia had progressed compared with the previous period. (C) Chest CT on the 5th week of admission showed that bilateral pneumonia had absorbed compared with the previous period.

**Figure 3.**
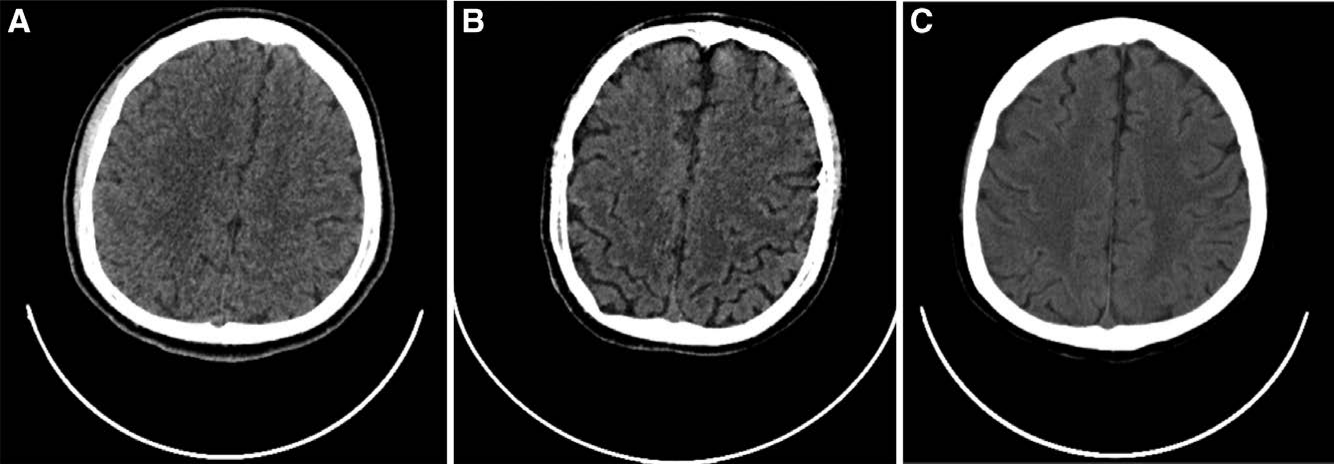
(A) Head CT on admission showed no significant abnormality. (B) Head CT examination after 2 weeks in hospital showed no obvious abnormality. (C) Head CT examination 5 weeks after hospitalization revealed mild cerebral atrophy. CT = computed tomography.

An initial arterial blood gas analysis of the patient revealed a fraction of inspired oxygen (FIO_2_) of 33% (21–100), pH of 7.33 (7.35–7.45), partial pressure of oxygen (PaO_2_) of 116 mm Hg (80–100), partial pressure of carbon dioxide (PaCO_2_) of 32.5 mm Hg (35–45), chlorion (Cl^‐^) of 114 mmol/L (98–106), potassium ion (K^+^) 2.6 mmol/L (3.5–5.58), calcium ion (Ca^2+^) 1.12 mmol/L (1.15–1.29), bicarbonate (HCO_3_^‐^) of 18.5 mmol/L (22–26), lactic acid of 2.6 mmol/L (0.5–1.6), oxygenation index (pO_2_/FO_2_) of 379 (400–500), and standard base excess of ‐8.9 mmol/L. The results of serum biochemistry were the following (Tables 1 and 2): procalcitonin of 100 (0–0.05 ng/mL), interleukin-6 of 326.00 pg/mL (0–7), K^+^ of 3.36 mmol/L (3.5–5.3), Na^+^ of 141 mmol/L (137–147), Cl^‐^ of 104 mmol/L(99–110), Ca^2+^ of 2.44 mmol/L (2.08–2.6), brain natriuretic peptide > 35,000.00 pg/mL (0–125), myoglobin of 12,030 ng/mL (28–72), tropninI of 0.981 ng/mL (0.00–0.033), lipase of 96 U/L (0–60), amylase of 745 U/L (0–220), urinary amylase of 2006 U/L (0–1200). Cerebrospinal fluid biochemistry: Cl^‐^ of 109.20 mmol/L (120–132), protein of 468.20 mg/L (150–450), creatine kinase of 38 U/L (0–30), Ca^2+^ of 0.25 mmol/L (1–1.4), Mg^2+^ of 0.86 mmol/L (1.1–1.4), uric acid of 23.00 μmol/L (0–20). Cerebrospinal fluid was negative for *Cryptococcus neoformans* capsular antigen, cerebrospinal fluid lactic acid was not abnormal, cerebrospinal fluid smear did not find cryptococcus and acid-fast bacillus. Electrocardiogram on admission showed sinus tachycardia (Fig. 1). Computed tomography of the chest demonstrated bilateral pneumonia (Fig. 2) and head computed tomography showed mild cerebral atrophy (Fig. 3). Echocardiogram showed left ventricular (LV) ejection fraction of 45%, the LV systolic function was slightly decreased, the left atrium was slightly larger, the right atrium was full, and the overall systolic movement of the LV wall was slightly weakened in the resting state. Thyroid sonogram suggested diffuse thyroid disease, considering the possibility of hyperthyroidism. There were no significant abnormalities in the electroencephalogram and no definite epileptoid features.

**Table 1:** Thyroid function tests.

	Day 1 (admission)	Day 3	Day 6	Day 30	Day 59 (discharge)	Reference range
T3 (nmol/L)	2.87	1.36	1.34	0.58	1.27	1.34–2.75
T4 (nmol/L)	231.7	140	83.24	44.88	78.5	78.38–157.40
FT3 (pmol/L)	9.17	4.43	3.88	1.64	3.6	3.60–6.00
FT4 (pmol/L)	32.21	16.16	15.9	8.94	7.86	7.86–14.41
TSH (mIU/L)	0.01	1.01	0.01	0.01	1.64	0.34–5.65
TGAb (%)	81.88			80.7		<30
TRAb (IU/L)	0.631			0.38		0–15
TPOAb (IU/mL)	271			109		0–30

FT3 = free triiodothyronine, FT4 = free thyroxine, T3 = triiodothyronine, T4 = thyroxine, TGAb = anti-thyroglobulin antibodies, TPOAb = thyroid peroxidase antibody, TRAb = thyrotrophic receptor antibody, TSH = thyroid stimulating hormone.

**Table 2:** Laboratory findings.

	Day 1 (admission)	Day 3	Day 6	Day 18	Day 30	Day 50	Day 59 (discharge)	
WBC	6.75	12.29	17.48	19.43	16.01	12.06	8.82	3.5–9.5
HGB	156	94	95.1	87	80.5	102.5	99	130–175
NEU%	0.834	0.96	0.891	0.867	0.794	0.683	0.661	0.4–0.75
CRP	84.56	116.4	189.1	154.8	109.6	83.34	10	0–10 mg/L
TBiL	61.7	75	102.9	50.1	36.9	23.8	17.6	3.4–20.5 μmol/L
DBiL	13.9	40.2	77.1	44.2	29.8	19.3	7.8	0–6.8 μmol/L
AST	43	1295	648	608	256	67	39	15–45 U/L
ALT	99	979	305	200	77	32	41	9–60 U/L
UREA	5.64	26.8	51.86	72.51	60.94	43.55	18.6	2.9–8.2 mmol/L
CREA	108	360	689	486	574	261	104	59–104 μmol/L
UA	569	919	713	490	349	535	215	208–428 μmol/L
CK	118	5122	52,783	12,634	1952	429	62	38–174 U/L
CK-MB	16	191	1397	308	96	53	25	0–25 U/L

ALT = alanine aminotransferase, AST = aspartate aminotransferase, CK = creatine kinase, CREA = creatinine, CRP = C-reactive protein, DBiL = direct bilirubin, HGB = hemoglobin, NEU = neutrophil, TBiL = total bilirubin, UA = uric acid, WBC = white blood cell.

According to the patient’s manifestations of thyroid dysfunction, the Burch–Wartofsky scoring scale (BWPS) score was 90 (body temperature 25 + epilepsy 30 + nausea and vomiting 10 + tachycardia 25), which was highly suggestive of thyroid storm. After he was admitted to the hospital, he began to treat thyroid storm, including anti-hyperthyroidism by propylthiouracil, control of heart rate by propranolol, antagonism of stress by hydrocortisone and inhibition of peripheral effects of thyroid hormone, massive fluid rehydration, protection of liver and kidney, continuous renal replacement therapy, correction of electrolyte disorders, and other supportive treatments. The patient’s breathing was relatively fast, continuously at 45 to 50 times/minute. Repeated blood gas analysis showed that the oxygen partial pressure was significantly lower than before. Considering respiratory failure, endotracheal intubation and ventilator-assisted breathing were performed. The patient’s body temperature continued to rise, reaching a maximum of 40 °C, and did not decrease after proper cooling. Infection indexes such as leukocyte, neutrophil percentage, C-reactive protein, procalcitonin, and interleukin-6 were significantly elevated. Multidrug-resistant *Acinetobacter baumannii* was cultured in both sputum and bronchoalveolar lavage fluid. Therefore, piperacillin 5 g q8h, tigecycline 50 mg q12h (first dose 100 mg), and voriconazole 0.2g q12h anti-infection therapy was administered. On the 15th day of admission, he again developed staring eyes, rapid breathing, and accelerated heart rate, but no limb convulsions or foaming at the mouth. This was suspected to be an epileptic seizure, and he was given oxcarbazepine to control the epilepsy. In addition, the patient had an increase in creatine kinase of >5 times the upper limit of normal and was treated with methylprednisolone 0.25 g qd anti-inflammatory therapy considering rhabdomyolysis. After the above treatment, the patient’s thyroid function, myocardial enzymes, liver and kidney function gradually returned to normal (Tables 1 and 2). The patient’s vital signs stabilized, his consciousness level improved, and after being taken off the ventilator, he was transferred to the Department of Traditional Chinese Medicine, where he received Chinese medicine and rehabilitation treatment, and was discharged from the hospital after his condition improved.

## 3. Discussion

Thyroid storm is a rare acute hypermetabolic condition caused by excessive release of thyroid hormones. Common causes include infection, surgery, emotional stress, iodine load, noncompliance with medication, and other acute medical conditions.^[13–15]^ Studies have reported that the annual incidence of thyroid storm in the general community is 0.57 to 0.76 per 100,000 people, and in hospitalized patients, the annual incidence is 4.8 to 5.6 per 100,000 people.^[16]^ The mortality rate of hospitalized patients with thyroid storm is 10% to 30%, which is 12 times higher than that of patients with thyrotoxicosis.^[17,18]^ Without proper treatment, thyroid storms are almost always fatal. Due to the lag in laboratory tests, thyroid storm must be diagnosed in a timely manner based on clinical symptoms in order to provide life-saving treatment quickly. Therefore, a high degree of suspicion of an impending thyroid storm and the ability to diagnose it early are essential, which in turn depend on a thorough understanding of the typical and atypical clinical features of this disease. The diagnosis of thyroid storm was mostly based on the scoring scheme proposed by Bruch and Wartofsky in 1993, and the BWPS score ≥ 45 was considered as thyroid storm.^[1,19]^ However, in practice, it is prudent to start aggressive treatment as soon as impending thyroid storm is suspected. The patient in this case had a BWPS score of at least 90, confirming the diagnosis of thyroid storm.

Hyperthyroidism can be complicated by a variety of neurological diseases, such as thyrotoxic encephalopathy, acute hyperthyroidism myopathy, chronic hyperthyroidism myopathy, hyperthyroidism hypokalemic periodic paralysis, hyperthyroidism myasthenia gravis, but metabolic encephalopathy is rare.^[20,21]^ Metabolic encephalopathy refers to the brain dysfunction caused by metabolic changes in the brain tissue affected by various systemic diseases, and is a common complication in the treatment of other diseases.^[12]^ Metabolic encephalopathy is more common in intensive care units, especially in conditions such as liver and kidney failure, sepsis, electrolyte imbalance, or endocrine dysfunction.^[22]^ However, metabolic encephalopathy is rare in the course of hyperthyroidism, especially in the course of thyroid storm. To our knowledge, there have been 4 reports of thyroid storm complicated by metabolic encephalopathy worldwide and this is the 5th report of such a case.

Encephalopathy is usually multifactorial due to the interaction of different organ systems. The pathogenesis of thyroid storm complicated by metabolic encephalopathy is not yet clear, and the possible related mechanisms are speculated: the brain is an organ sensitive to thyroid hormone, and a large amount of thyroid hormone accelerates the oxidation process of brain cell mitochondria, consumes a lot of energy, and leads to hypoxia and energy deficiency of nerve cells.^[23]^ Thyroid hormone can up-regulate the Na^+^ current density, make the sympathetic nerve overexcited, increase the concentration of catecholamines in the blood, resulting in small artery spasm and increased blood viscosity, thus affecting the blood supply to the brain.^[24–26]^ Thyroid hormone is a lipophilic hormone, easy to damage brain tissue through the blood–brain barrier, can increase the excitability of the central nervous system, or due to hypermetabolism, thyroid hormone metabolites directly cause brain damage. The hypermetabolic state of thyroid storm accelerates the depletion of thiamine reserves and thus causes encephalopathy.^[27,28]^ During thyroid storm, thyroid-related antibodies may increase and bind to the antigen targets of thyroid autoantibodies distributed in the brain, causing the resident immune cells (microglia) in the brain to be activated and release a variety of proinflammatory cytokines, thereby causing severe inflammatory responses in the brain.^[10,29,30]^ Thyroid storm leads to systemic multiple organ failure and electrolyte disorder, while changes in the internal environment can affect the metabolism of intracranial water and electrolyte, resulting in metabolic disorders of intracranial brain tissue.^[31,32]^ When brain thyroid metabolism is disrupted, key brain neurotransmitter systems are affected, leading to adverse neuropsychiatric outcomes. The failure of the brain balance mechanism caused by the synergistic effect of thyrotoxicosis and systemic injury may lead to an inappropriate elevation of T3 activity in the brain.^[33]^ Intense stress stimulation can relieve central sympathetic nervous system inhibition associated with thyrotoxicosis and aggravate tissue hyperresponsiveness to β-adrenergic stimulation directly induced by thyroid hormone.^[34]^ The inflammation that occurs during thyroid storms may play a key role in driving brain thyroid hormone imbalances and their interaction with neurotransmitter circuits.^[23]^ Therefore, multiple mechanisms may lead to the occurrence of thyroid storm complicated by metabolic encephalopathy.

In our case, metabolic encephalopathy was closely associated with thyroid storm. The clinical manifestations of thyroid storm complicated by metabolic encephalopathy are not specific, and there is no uniform standard for diagnosis. The evidence of clinical and laboratory diagnosis of hyperthyroidism in this case is as follows: a history of irregular use of antithyroid drugs; antithyroid therapy is effective; specific drug toxic encephalopathy, carbon monoxide toxic encephalopathy, neuro-immune system disease, paraneoplastic syndrome and other related brain lesions were excluded. Nervous system diseases are closely related to endocrine-metabolic diseases. A Polish team has shown in a large number of studies that patients with thyroid dysfunction, even in the euthyroid phase, experience both disturbances in brain bioelectrical activity and changes in brain metabolism, as confirmed by neuroimaging studies.^[35]^ Most likely, these are the reason of many non-endocrine manifestations of the disease and prove that normal thyroid function is not an indicator of the body’s balance and that there is a risk of developing encephalopathy.

We summarized the imaging features, clinical manifestations, related treatment and prognosis of the published cases of thyroid storm complicated with metabolic encephalopathy in Table 3. The clinical symptoms and imaging features of thyroid storm complicated with metabolic encephalopathy are diverse. The central nervous system damage mainly manifests as varying degrees of consciousness disorders such as acute mental confusion state, decortication state, epileptic seizures, bulbar palsy, pyramidal tract involvement, spinothalamic tract involvement, and extrapyramidal system involvement. The 5 patients all had tachycardia and changes in consciousness, but the severity was not the same, the mild one only had drowsiness, the severe one may have seizures and coma. Imaging features vary from mild brain swelling and atrophy to frontal lobe and thalamic lesions. Patients 1, 2, and 3 were all considered to be thyroid storm complicated with Wernicke encephalopathy. Wernicke encephalopathy is a rare metabolic encephalopathy originally seen in patients with alcoholism, resulting from thiamine deficiency causing confusion, ataxia, and nystagmus.^[36–38]^ However, the patients 1, 2, and 3 had no history of alcoholism. The triggering causes were hyperemesis gravidarum and malnutrition, which led to thiamine deficiency in the hyperthyroidism hypermetabolic state, thus causing the occurrence and development of thyroid storm complicated with Wernicke encephalopathy. The clinical symptoms of patients 4 and 5 are similar, without the typical triad of Wernicke encephalopathy, but they both have seizures and coma, and both require extracorporeal systemic treatment. Among the 5 patients summarized in this paper, patients 1, 2, 3, and 4 had no previous history of hyperthyroidism, and the patients were treated in time mainly by the clinicians’ experience and high vigilance. Except for patient 2 who was left with significant neurological deficits, the other 4 patients recovered well. Thyroid storm complicated by metabolic encephalopathy requires early diagnosis and treatment, and the neurological function damage caused by it is usually reversible. If the primary disease is not treated in time, the brain tissue may continue to be damaged, and the development of the disease is often irreversible. Therefore, when patients with hyperthyroidism present with neurological symptoms and signs or imaging changes in clinical practice, we should consider differentiating them from thyroid storm complicated by metabolic encephalopathy and provide timely treatment.

**Table 3:** Summary of cases of thyroid storm complicated by metabolic encephalopathy.

Patient	1^[7]^	2^[6]^	3^[5]^	4^[4]^	5 (present case)
A (Y)/G	27 F	39 M	36 F	23 M	20 M
Inducement	Hyperemesis gravidarum	Malnutrition	Hyperemesis gravidarum	Alcoholism	Discontinuation of anti thyroidal medications
Course of hyperthyroidism (m)	0	0	0	0	24
Nervous system symptoms	Coma, nystagmus, ataxia	Acute confusion and somnolence, nystagmus, ataxia	Delirium, lethargy, nystagmus, ataxia	Seizures, coma	Seizures, coma
Cardiovascular symptoms	Tachycardia	Tachycardia	Tachycardia	Nodal tachycardia	Supraventricular tachycardia
BWPS score	70	55	65	85	90
Thyroid function at the time of metabolic encephalopathy (reference ranges are in parentheses)	FT3 8. 26 pg/mL (4–5.8)	FT3 17.47 pg/mL (2.57–4.43)	T3 resin uptake was 35% (22.5–37%)	FT3 29.20 pmol/L (3.6–7.0)	FT3 9.17 pmol/L (3.6–6.0)
	FT4 2.77 ng/dL (1.03–2.21)	FT4 7.62 ng/dL (0.93–1.70)	FT4 4.58 (1.4–3.8)	FT4 65.30 pmol/L (11.3–22.5)	FT4 32.21 pmol/L (7.86–14.41)
	TSH 0.02 IU/mL (0.55–4.8)	TSH 0.01 IU/mL (0.27–4.20)	TSH < 0.01 mIU/mL (0.55–4.78)	TSH 0.001 μIU/mL (0.35–1)	TSH 0.01 mIU/L (0.34–5.65)
Thiamine blood level	NA	0.7 μg/dL (1.6–4.0 μg/dL)	NA	NA	NA
Lumbar puncture	Normal	Normal	None	None	Normal
Imaging findings	Slight swelling of the brain	The mammillary bodies, fornix, medial thalami, peri-aqueductal midbrain, superior vermis, and bilateral precentral gyri lesions	Hyperintense abnormal signals in both thalami and the periaqueductal gray matter of midbrain	Bilateral frontal lobe lesions	Mild cerebral atrophy
EEG	Periodic diffuse slow wave activities involving the whole brain	None	Normal	None	Normal
Antithyroid medications	Thiamazole	Propylthiouracil	Methimazole	Propylthiouracil and iodine	Propylthiouracil
Blood purification	None	None	None	2 plasma exchanges, 6 hemoperfusion cycles	13 CRRT
	Vitamin B1 250 mg i.v.	Thiamine	Thiamine 500 mg	NA	None
Outcome	Recovery	Significant neurological deficits	Recovery	Recovery	Recovery

BWPS = Burch–Wartofsky scoring scale, CRRT = continuous renal replacement therapy, EEG = electroencephalogram, FT3 = free triiodothyronine, FT4 = free thyroxine, NA = not available, TSH = thyroid stimulating hormone.

The treatment of thyroid storm mainly includes inhibition of excess thyroid hormone synthesis and release by antithyroid drugs and iodine. In addition, β-blockers and glucocorticoids can also be used in the clinical treatment of hyperthyroid crises. However, studies have found that routine use of glucocorticoids does not improve survival in thyroid storm patients, suggesting that clinicians should individualize the use of glucocorticoids, taking into account the risk of infection and hyperglycemia.^[39]^ For Wernicke encephalopathy, timely and appropriate thiamine supplementation is necessary. For patients who cannot tolerate drugs or who have failed drug therapy, alternative treatments need to be considered, including the use of extracorporeal systems such as continuous renal replacement therapy, veno-arterial extracorporeal membrane oxygenation, and therapeutic plasma exchange.^[17,40–42]^ Successful treatment of thyroid storm depends on early inhibition of thyroid hormone secretion and suppression of peripheral T4 to T3 transformation. After treatment, the thyroid hormone levels of patients with thyroid storm and encephalopathy returned to normal, and cranial imaging showed that the brain lesions also returned to normal.^[4]^ With the improvement of hyperthyroidism, the patient’s nerve damage is reversible, suggesting that the treatment of thyroid storm is the key to improve the condition.

Our study has following limitations. First, the number of cases we screened by BWPS score was small, and some data was insufficient in those cases, so we could not make a stricter description and explanation on some results like blood tests, lumbar puncture, and electroencephalogram. Second, the efficacy and route of administration of anti-hyperthyroidism and anti-encephalopathy drugs in these patients are not fully understood. Therefore, the method required to control thyroid storm complicated by metabolic encephalopathy (thiamine vs anti-hyperthyroidism drugs), and how long anti-hyperthyroidism drugs should be used require further study.

## 4. Conclusions

Metabolic encephalopathy is a devastating complication of thyroid storm, which can be caused by acute and chronic metabolic brain damage. With the established severe morbidity and mortality of thyroid storm, it is crucial that emergency physicians quickly recognize and manage this pathology. In addition to the medical treatment to control sympathomimetic symptoms, inhibit synthesis and release of thyroid hormones, as well as supportive measures for systemic decompensation, predisposing factors for thyroid storm should be corrected. This is an expanding area of research that promises to lead to better understanding, treatment and patient outcomes.

## Acknowledgments

The authors are very grateful to the patient for his kind contribution to this study.

## Author contributions

**Resources:** Bixiu Ban.

**Writing – original draft:** Yuping Liu.

**Writing – review & editing:** Zuojie Luo.
